# Knowledge and practices related to hepatitis A among adolescents: a cross-sectional study in Curitiba, Paraná, 2024

**DOI:** 10.1590/S2237-96222026v35e20250871.en

**Published:** 2026-03-16

**Authors:** Janilza Silveira Silva, Renata Barbosa Vilaça Marques de Carvalho, Alcides Souto de Oliveira, Tatiane Bartneck Telles, Liza Regina Bueno Rosso, Leia Regina Silva, Diego Spinoza dos Santos, Dirlene Pacheco Venâncio, Monique Boese, Katiuska Ferraz Jansen Negrello, Cláudia Weingaertner Palm, Clea Elisa Ribeiro, Aroldo José Borges Carneiro, Silvio Luis Rodrigues Almeida

**Affiliations:** 1 Ministério da Saúde, Programa de Treinamento em Epidemiologia Aplicada aos Serviços do SUS, Brasília, DF, Brazil; 2 Centro de Epidemiologia da Secretaria Municipal da Saúde de Curitiba, Paraná, PR, Brazil; 3 Prefeitura de Curitiba, Secretaria Municipal da Saúde, Curitiba, Paraná, PR, Brazil; 4 Centro de Informações Estratégicas em Vigilância em Saúde de Curitiba, Paraná, PR, Brazil

**Keywords:** Hepatitis, Hepatitis A Virus, Adolescents, Health-Related Behaviors, Health Knowledge, Attitudes, Practice, Hepatitis, Virus de la Hepatitis A, Adolescentes, Comportamientos Relacionados con la Salud, Conocimientos, Actitudes y Práctica en Salud

## Abstract

**Objective:**

To assess the knowledge and practices of adolescents attending state public schools in Curitiba, Paraná, regarding hepatitis A.

**Methods:**

A cross-sectional study evaluating the knowledge and practices of adolescents aged 16 years or older, recruited by convenience sampling from public schools in Curitiba, Paraná. Knowledge was assessed using multiple-choice questions, organized into three dimensions: Pathology; Transmission and Symptomatology; and Prevention and Cure. Risk behaviors were scored in ascending order of exposure and grouped into five dimensions: Hygiene; Dietary Habits; Drinking Water; Tobacco Products; and Sexual Behaviors. Scores were standardized on a 0-10 scale and classified as very low (0⊢2.5), low (2.5⊢5), moderate (5⊢7.5), and high (7.5⊢10). Appropriate descriptive and inferential analyses were performed.

**Results:**

A total of 298 adolescents participated (52.0% female; 63.1% self-identified as White), with a mean age of 16.6±0.7 years. The mean knowledge score was 3.7±3.0 and was higher among females (p=0.039). The lowest mean score was observed in the Transmission and Symptomatology dimension (3.3±3.2; p<0.001). The mean risk behaviors score was 1.7±1.3 and was higher among males (p=0.001); higher scores indicate greater exposure to risk behaviors. The Sexual Behaviors dimension presented the highest score (6.3±1.4; p<0.001). No correlation was found between knowledge and risk behaviors (Spearman’s rho=0.00; p=0.983).

**Conclusion:**

Knowledge about hepatitis A was low, particularly regarding transmission and symptoms. Although the overall risk behaviors score fell within the low category, the Sexual Behaviors dimension was classified as moderate, indicating a relevant vulnerability that requires targeted interventions for adolescents.

## Resumen

**Objetivo:**

Evaluar los conocimientos y las prácticas de los adolescentes de las escuelas del estado de Curitiba, Paraná, con respecto a la hepatitis A.

**Métodos:**

Estudio transversal que evalúa los conocimientos y las prácticas de los adolescentes ≥16 años, seleccionados mediante muestreo de conveniencia en las escuelas públicas de Curitiba, Paraná. El conocimiento se evaluó mediante preguntas de elección múltiple, organizadas en tres dimensiones: Patología; Transmisión y Sintomatología; Prevención y Cura. Las prácticas de riesgo se calificaron en orden ascendente de exposición y se agruparon en cinco dimensiones: Higiene; Hábitos Alimenticios; Agua para Consumo; Productos Fumígenos; y Prácticas Sexuales. Las puntuaciones se estandarizaron en una escala de 0 a 10 y se clasificaron como muy baja (0⊢2,5), baja (2,5⊢5), moderada (5⊢7,5) y alta (7,5⊢10). Se utilizaron análisis descriptivos e inferenciales apropiados.

**Resultados:**

Participaron 298 adolescentes (52,0% del sexo femenino; 63,1% autodeclarados blancos), con una edad media de 16,6 ± 0,7 años. La puntuación media de conocimiento fue de 3,7±3,0, mayor entre las niñas (p-valor 0,039). La media más baja se produjo en la dimensión Transmisión y Sintomatología (3,3±3,2; p-valor <0,001). La puntuación media de las prácticas de riesgo fue de 1,7±1,3, mayor en el sexo masculino (p-valor 0,001). Las puntuaciones más altas corresponden a una mayor exposición a prácticas de riesgo. La dimensión Prácticas Sexuales tuvo la puntuación más alta (6,3±1,4; p-valor p<0,01). No hubo correlación entre el conocimiento y las prácticas de riesgo (correlación de Spearman rho=0,00; p-valor 0,983).

**Conclusión:**

El conocimiento sobre la hepatitis A fue bajo, especialmente en lo que respecta a la transmisión y los síntomas. Aunque la puntuación general de las prácticas de riesgo fue baja, la dimensión Prácticas Sexuales presentó una clasificación moderada, lo cual muestra una vulnerabilidad relevante que requiere intervenciones específicas para adolescentes.

**Ethical aspects**

This research respected the ethical principles, having obtained the following approval data:

Research Ethics Committee: National Research Ethics Committee

Opinion number: 7.545.013

Approval date: 13/6/2025

Certificate of Submission to Ethical Appraisal: 87956525.0.0000.0008

Informed consent: Dismissed.

## Introduction

Hepatitis A is an infectious liver disease caused by the Hepatitis A virus (HAV), transmitted mainly via the fecal-oral route, either by direct contact with infected individuals or the ingestion of contaminated water and food ^1^. Although it may cause outbreaks and lead to severe cases of fulminant hepatitis, especially in adults, infection in children and adolescents tends to be milder or asymptomatic ^2^. In Brazil, the hepatitis A vaccine was introduced into the Brazilian National Childhood Immunization Schedule in 2014, with a single dose administered at 15 months of age (and applicable from 12 months to 4 years, 11 months, and 29 days), representing an important milestone in disease control ^3^.

The city of Curitiba, in Paraná State, experienced a hepatitis A outbreak from late 2023 to mid-2024, including reported cases among school-aged children and adolescents ^4^. The occurrence of cases in this age group, even in the context of an established childhood vaccination program, highlighted the relevance of understanding levels of information about hepatitis A and the behaviors adopted in relation to the disease, particularly within the school environment. 

Studies exploring population-level knowledge of communicable diseases, as well as behaviors related to prevention and health care, are widely used in public health. This approach makes it possible to identify knowledge gaps and behaviors that may favor the spread of infectious agents and is especially useful in outbreaks or among vulnerable populations ^5^. Regarding viral hepatitis, investigations conducted in various countries and populations have demonstrated heterogeneous levels of knowledge and the persistence of inappropriate behaviors, even among individuals with some degree of information about the disease ^6^ ^-^ ^9^. 

In this context, this study aimed to assess the knowledge and practices of adolescents attending state public schools in Curitiba, Paraná, regarding hepatitis A.

## Methods

### Study design

This is a cross-sectional study conducted between July 3 and 4, 2024, including five state public schools in the city of Curitiba, Paraná (School A, School B, School C, School D, and School E). 

### Context

From November 1, 2023, to May 29, 2024, a total of 281 cases of hepatitis A were confirmed in Curitiba, Paraná. Initial cases were concentrated among young adults; however, as the outbreak progressed, cases were observed among school-aged children and adolescents, expanding the affected age profile. In this context, the investigation was conducted by a team from the Field Epidemiology Training Program (EpiSUS-Avançado) of the Brazilian Unified Health System (SUS), in partnership with the local surveillance team.

### Participants

The study was conducted with adolescents aged 16 years or older who were enrolled in the five selected state public schools in Curitiba, Paraná. Eligibility criteria were as follows:

- Inclusion: Participants aged 16 years or older who were regularly enrolled and who provided informed consent to participate in the study.
- Exclusion: Adolescents unable to understand the questionnaire due to cognitive capacity, as assessed and reported by school staff.

After schools identified eligible students (≥16 years) and informed consent was obtained, participants were directed to a reserved room at their respective schools, where they received final instructions and completed the questionnaire in a self-administered and anonymous format using tablet computers provided by the research team, ensuring privacy and confidentiality of responses.

### Variables

The questionnaire included sociodemographic variables, knowledge variables, and behavior variables related to hepatitis A. Sociodemographic variables included sex, age, race/skin-color, and school of origin. Knowledge variables were organized into three dimensions: i) Pathology - having heard of hepatitis A, type of disease (communicable or not), causative microorganism, and main affected organ; ii) Transmission and symptomatology - mode of transmission, signs and symptoms, and risk exposures; iii) Prevention and cure - vaccine availability, forms of prevention, possibility of cure, and prior hepatitis A infection. Practice variables were grouped into five dimensions: i) Hygiene - handwashing habits and sharing of personal objects or utensils; ii) Dietary habits - consumption of raw or undercooked foods; iii) Drinking water - source and treatment of water, as well as behaviors related to the use of school drinking fountains; iv) Tobacco products - use of cigarettes or other tobacco products; v) Sexual behaviors - sexual initiation, partner characteristics, condom use, oro-anal sexual practices, anal intercourse (with or without a condom), and anal penetration with fingers or objects.

The questionnaire was pretested with local surveillance technicians prior to its application during the survey. The investigators chose to focus on the Knowledge and Practice dimensions, which offer greater objectivity and reproducibility, allowing for a more consistent assessment of behavior related to hepatitis A. The Attitudes dimension was not analyzed because it is a subjective construct that is difficult to measure in self-administered surveys, particularly among adolescents.

### Data sources and measurement

Data were collected using a self-administered semi-structured questionnaire made available on the REDCap platform. The questionnaire was developed based on studies of knowledge, attitudes, and practice related to viral hepatitis ^6^ ^-^ ^10^ and on the Brazilian Health Surveillance Guide. Participant recruitment was conducted through the selected schools.

For the estimation of knowledge scores, each correct answer was worth 1 point. Scores for each dimension were converted to a 0-10 decimal scale, and the total score was similarly standardized by dividing the total number of correct answers by the maximum possible score and multiplying by 10. The distribution of points by knowledge dimension is detailed in the “data availability” section. Practice scores were assigned in ascending order of risk, and both the dimension-specific scores and the total score, referred to as risk behaviors, were converted to a 0-10 scale, in which higher scores indicated greater risk. Scores by practice dimension are detailed at the link provided in the “data availability” section. 

The classification of both knowledge scores and risk behavior scores followed the categories: very low (0 ⊢ 2.5), low (2.5 ⊢ 5), moderate (5 ⊢ 7.5), and high (7.5 ⊢ 10). Higher scores for behaviors indicate greater risk. Scores were estimated using the following equation:

$$Score=\frac{Total\;score\;on\;the\;knowledge\;test}{Maximum\;possible\;score\;on\;the\;test}\times 10$$

### Biases

Potential sources of bias considered included selection, information, and classification bias. Selection bias may have occurred due to convenience sampling and the pre-holiday period, which reduced student attendance at schools and limited the generalizability of the findings. Information bias is inherent to self-administered questionnaires that include sensitive content and may result in social desirability bias (overestimation of knowledge and underestimation of risk behaviors); this was mitigated by ensuring anonymity. 

### Study size

In total, five schools were selected by convenience: four because they had recorded confirmed hepatitis A cases from November 1, 2023, to May 29, 2024, and one (School E), with no confirmed cases, was included for comparison. School E is located in a health district in the southern region of Curitiba characterized by less favorable social and environmental indicators, particularly regarding water and sanitation ^11^. 

The estimated sample size was 284 students, assuming an expected frequency of 50% and a 5% margin of error, based on a population of 1,090 students aged 16 years or older. Participants were recruited by convenience sampling, and all eligible students were invited to participate.

### Statistical methods

Descriptive analyses were performed, and comparisons between groups were conducted using Student’s t-test, Mann-Whitney test, and Kruskal-Wallis test, with adjustment for multiple comparisons using the Bonferroni method. Correlations between continuous variables were assessed using Spearman’s correlation coefficient. A 5% significance level was adopted (p-value<0.05). Statistical analyses were conducted using R programming language (version 4.4.0). 

## Results

A total of 300 questionnaires were submitted, of which 298 were completed and included in the analysis. Most participants were female (52.0%) and self-identified as White (63.1%), with a mean age of 16.6±0.7 years (Table 1).

**Table 1:** Demographic characteristics of participants in the study on knowledge and practices regarding hepatitis A. Curitiba, Paraná, 2024 (n=298)

Characteristic	n (%)
Sex	
Female	153 (52.0)
Male	141 (48.0)
Ethnicity/skin color	
White	183 (63.1)
Mixed-race	76 (26.2)
Black	22 (7.6)
Yellow	5 (1.7)
Indigenous	4 (1.4)
School	
School A	50 (16.8)
School B	73 (24.5)
School C	47 (15.8)
School D	42 (14.1)
School E	73 (24.5)

Knowledge assessment showed that although a substantial proportion of adolescents (70.1%) had heard of hepatitis A, specific knowledge about the disease was limited (Table 2). 

The overall mean knowledge score for hepatitis A was 3.7±3.0, corresponding to a low level of knowledge. Higher scores were observed among female students (p-value 0.039) (Table 3). No statistically significant differences in knowledge scores were found between schools (p-value 0.258).

The Pathology dimension showed better results compared with the Transmission and Symptomatology dimension (p-value<0.001), as shown in Table 4. The lowest proportions of correct answers were observed for questions related to risk exposures, preventive measures, and signs and symptoms of the disease.

**Table 2:** Specific knowledge about hepatitis A among school adolescents by theme, question, and correct answer. Curitiba, Paraná, 2024 (n=298)

Theme	Abbreviated question	Correct answer (%)
General knowledge	Have you heard of hepatitis A?	Yes (70.1)
Transmissibility	Type of disease	Communicable (43.6)
Etiological agent	Caused by	Virus (27.9)
Mode of infection	Main route	Fecal-oral transmission (39.9)
Affected organ	Which organ?	Liver (30.2)
Vaccine	Is there a vaccine?	Yes (46.6)
Risk exposure	Contaminated water/food	Yes (36.2)
	Unprotected oro-anal sexual practices	Yes (35.2)
Signs/symptoms	Fever	Yes (42.3)
	Yellow eyes	Yes (35.2)
Prevention	Hand hygiene	Yes (39.9)
Cure	Is it curable?	Yes (40.3)

**Table 3:** Knowledge scores on hepatitis A by sex, school, and knowledge dimensions, presented as mean (±SD) and median (IQR). Curitiba, Paraná, 2024 (n=298)

Score	Mean (±SD)	Median (IQR)	p-value
Overall	3.7 (±3.0)		
Sex			
Female	4.0 (±3.1)	4.7 (6.5)	0,039^a^
Male	3.3 (±2.9)	4.1 (5.9)	
School			
School C	4.3 (±2.9)	4.7 (4.4)	0,258^b^
School D	4.0 (±3.1)	4.7 (5.9)	
School E	3.8 (±2.9)	4.7 (6.5)	
School B	3.8 (±3.0)	4.1 (6.5)	
School A	2.9 (±2.9)	2.7 (4.7)	
Knowledge dimension			
Pathology	4.3 (±3.5)	5.0 (7.5)	0,004^c^
Transmission and symptomatology	3.3 (±3.2)	3.8 (6.3)	
Prevention and cure	3.8 (±3.5)	4.0 (6.0)	

^a^ Student’s t-test; ^b^ANOVA; ^c^Kruskal-Wallis test.

**Table 4:** Risk behavior scores related to hepatitis A by sex, school, and practice dimensions, presented as mean (±SD). Curitiba, Paraná, 2024 (n=298)

Score	Mean (±SD)	p-value
Overall	1.7 (±1.3)	
Sex		
Male	1.9 (±1.5)	0.001^a^
Female	1.4 (±0.9)	
School		
School D	2.1 (±1.4)	0.016^b^
School B	1.8 (±1.4)	
School C	1.7 (±1.3)	
School A	1.6 (±1.1)	
School E	1.3 (±1.1)	
Practice dimension		
1. Hygiene	2.7 (±1.5)	<0.001^b^
2. Dietary habits	3.9 (±4.4)	
3. Tobacco products	1.3 (±1.3)	
4. Drinking water	2.5 (±3.4)	
5. Sexual behavior	6.3 (±1.4)	

^a^ Student’s t test; ^b^Kruskal-Wallis test.

Regarding risk behaviors, the mean score was 1.7±1.3, classified as very low. Male students presented higher risk scores (p-value 0.001). Higher scores were observed particularly in the Sexual Behaviors and Dietary Habits dimensions. A significant difference was found between schools (p-value 0.016), specifically between School E and School D (p-value 0.010; Mann-Whitney U test) (Table 4). 

No correlation was observed between knowledge scores and risk behavior scores (Spearman’s rho=0.00; p-value 0.983).

## Discussion

The findings indicate that, although most adolescents attending the evaluated schools are aware of hepatitis A, there is a knowledge gap, particularly regarding mechanisms of transmission and identification of signs and symptoms. This knowledge profile contrasts with a predominantly low level of risk behaviors, suggesting that preventive behavior in the study population may be mediated by daily habits and social and environmental factors rather than by informed decision-making about the disease. 

Among the strengths of the study is the opportunity to collect data in the context of a recent outbreak, using a detailed and standardized instrument. Nevertheless, some limitations should be acknowledged. Convenience sampling of five schools and the inclusion of only adolescents aged 16 years or older limit the generalizability of the findings to all schools in Curitiba. Data collection during the pre-holiday period may have reduced participation, constituting a potential source of selection bias. Moreover, self-reporting may have introduced social desirability bias, although this was mitigated by anonymous questionnaire completion. Notably, no adjustment was made for a possible clustering effect by school, and that the “Attitudes” component was not included in the instrument, restricting the analysis to knowledge and practices and limiting understanding of the underlying behavioral determinants.

The mean risk behavior score was classified as very low, which may reflect both genuinely safer behaviors (low exposure to risk) and potential social desirability bias in responses. However, the Sexual Behaviors dimension showed higher scores compared with the other dimensions. In the context of hepatitis A, this finding is epidemiologically relevant, particularly with respect to oro-anal sexual practices and related personal hygiene behaviors, which are recognized as potential routes of fecal-oral transmission. 

Female adolescents presented significantly higher knowledge scores than males, a pattern consistent with the literature ^12^ ^,^ ^13^. Despite this difference, the overall low level of knowledge aligns with findings from studies on viral hepatitis among adolescents and young adults ^6^ ^,^ ^7^ ^,^ ^10^. The finding of higher risk behaviors among males is also consistent with previous studies reporting a higher prevalence of risk behaviors among boys, particularly to inconsistent use of condom and earlier sexual initiation ^14^. The lack of correlation between knowledge and risk behaviors has likewise been reported in other Knowledge, Attitudes, and Practices studies, indicating that information alone may be insufficient to modify behaviors-especially among adolescents, whose attitudes are strongly influenced by peers, cultural norms, and psychosocial factors ^15^ ^,^ ^16^. Overall, the occurrence of hepatitis A cases among adolescents during the outbreak in Curitiba, Paraná, combined with high risk scores in the sexual behavior domain in this age group, points to a vulnerability that warrants attention, considering not only the risk of hepatitis infection but also other sexually transmitted infections, such as human immunodeficiency virus, which is increasing among young people ^17^.

This study identified significant gaps in knowledge about hepatitis A among school-aged adolescents, as well as the presence of elevated risk behaviors in the domain of sexuality. These findings highlight the importance of considering both information levels and health-related behaviors when planning interventions targeted at this age group.

## Data Availability

The anonymized databases used in this study are publicly available in the SciELO Data repository, linked to the journal Epidemiologia e Serviços de Saúde (RESS), and can be accessed at: https://doi.org/10.48331/10.48331/SCIELODATA.6J5S6L Use of the dataset should be accompanied by citation as indicated in the repository.
