# Supplementary material for: Knowledge and practices related to hepatitis A among adolescents: a cross-sectional study in Curitiba, Paraná, 2024
Source: Epidemiol Serv Saude. 2026 Mar 16;35:e20250871. doi: 10.1590/S2237-96222026v35e20250871.en (PMC12995282; doi:10.1590/S2237-96222026v35e20250871.en)
Supplement: Translation

# Translation: Conhecimentos e práticas sobre a hepatite A em adolescentes: estudo transversal em Curitiba, Paraná, 2024

**DOI:** 10.1590/S2237-96222026v35e20250871.pt

**Authors:** Janilza Silveira Silva, Renata Barbosa Vilaça Marques de Carvalho, Alcides Souto de Oliveira, Tatiane Bartneck Telles, Liza Regina Bueno Rosso, Leia Regina Silva, Diego Spinoza dos Santos, Dirlene Pacheco Venâncio, Monique Boese, Katiuska Ferraz Jansen Negrello, Cláudia Weingaertner Palm, Clea Elisa Ribeiro, Aroldo José Borges Carneiro, Silvio Luis Rodrigues Almeida

**Affiliations:** 1 Ministério da Saúde, Programa de Treinamento em Epidemiologia Aplicada aos Serviços do SUS, Brasília, DF, Brasil; 2 Centro de Epidemiologia da Secretaria Municipal da Saúde de Curitiba, Paraná, PR, Brasil; 3 Prefeitura de Curitiba, Secretaria Municipal da Saúde, Curitiba, Paraná, PR, Brasil; 4 Centro de Informações Estratégicas em Vigilância em Saúde de Curitiba, Paraná, PR, Brasil

**Keywords:** Hepatites, Vírus da Hepatite A, Adolescentes, Comportamentos Relacionados com a Saúde, Conhecimentos, Atitudes e Prática em Saúde

## Abstract

**Objetivo:**

Avaliar os conhecimentos e práticas dos adolescentes de escolas estaduais de Curitiba, Paraná, quanto à hepatite A.

**Métodos:**

Estudo transversal avaliando conhecimentos e práticas de adolescentes ≥16 anos, recrutados por amostragem de conveniência em escolas públicas de Curitiba, Paraná. Conhecimento foi avaliado através de questões de múltipla escolha, organizadas em três dimensões: Patologia; Transmissão e Sintomatologia; Prevenção e Cura. Práticas de risco foram pontuadas em ordem crescente de exposição e agrupadas em cinco dimensões: Higiene; Hábitos Alimentares; Água para Consumo; Produtos Fumígenos; Práticas Sexuais. Pontuações foram padronizadas numa escala de 0 a 10 e classificadas como muito baixo (0⊢2,5), baixo (2,5⊢5), moderado (5⊢7,5) e alto (7,5⊢10). Utilizaram-se análises descritivas e inferenciais apropriadas.

**Resultados:**

Participaram 298 adolescentes (52,0% do sexo feminino; 63,1% autodeclarados brancos), com média de idade de 16,6 ± 0,7 anos. O escore médio de conhecimento foi 3,7±3,0, maior entre meninas (p-valor 0,039). A menor média ocorreu na dimensão Transmissão e Sintomatologia (3,3±3,2; p-valor<0,001). O escore médio de práticas de risco foi 1,7±1,3, superior no sexo masculino (p-valor 0,001). Escores mais altos correspondem a maior exposição a práticas de risco. A dimensão Práticas Sexuais apresentou o maior escore (6,3±1,4; p-valor<0,001). Não houve correlação entre conhecimento e práticas de risco (correlação de Spearman rho=0,00; p-valor 0,983).

**Conclusão:**

O conhecimento sobre hepatite A foi baixo, sobretudo quanto à transmissão e sintomas. Embora o escore geral de práticas de risco se enquadre na categoria baixa, a dimensão Práticas Sexuais apresentou classificação moderada, evidenciando vulnerabilidade relevante que requer intervenções específicas para adolescentes.

**Aspectos éticos**

Esta pesquisa respeitou os princípios éticos, obtendo os seguintes dados de aprovação:

Comitê de ética em pesquisa: Comissão Nacional de Ética em Pesquisa

Número do parecer: 7.545.013

Data de aprovação:13/6/2025

Certificado de apresentação de apreciação ética: 87956525.0.0000.0008

Registro do consentimento livre e esclarecido: Dispensado.

## Introdução

A hepatite A é uma doença infecciosa do fígado causada pelo Vírus da Hepatite A (HAV), transmitida principalmente pela via fecal-oral, seja por contato direto com pessoas infectadas ou pelo consumo de água e alimentos contaminados ^1^. Embora possa causar surtos e levar a casos graves de hepatite fulminante especialmente em adultos, em crianças e adolescentes a infecção tende a ser mais branda ou assintomática ^2^. No Brasil, a vacina contra a hepatite A foi introduzida no Calendário Nacional de Vacinação infantil em 2014, com uma dose aos 15 meses de idade (podendo ser utilizada a partir dos 12 meses até os 4 anos, 11 meses e 29 dias), o que constituiu um marco importante para o controle da doença ^3^.

A cidade de Curitiba, Paraná, enfrentou um surto de hepatite A do final de 2023 a meados de 2024, incluindo a notificação de casos em crianças e adolescentes em idade escolar ^4^. A ocorrência de casos nesse grupo etário, mesmo em um contexto de vacinação infantil estabelecida, evidenciou a relevância de compreender o nível de informação sobre a hepatite A e as práticas adotadas diante da doença, particularmente no ambiente escolar. 

Estudos que exploram o nível de informação de grupos populacionais sobre doenças transmissíveis, bem como práticas relacionadas à prevenção e ao cuidado em saúde, são amplamente utilizados em saúde pública. Esse tipo de abordagem permite identificar lacunas de conhecimento e comportamentos que podem favorecer a disseminação de agentes infecciosos, sendo especialmente útil em cenários de surtos ou em populações vulneráveis ^5^. No campo das hepatites virais, investigações realizadas em diferentes países e populações têm demonstrado níveis heterogêneos de conhecimento e a persistência de práticas inadequadas, mesmo entre indivíduos com algum grau de informação sobre a doença ^6^ ^-^ ^9^. 

Diante desse cenário, o estudo teve como objetivo avaliar os conhecimentos e práticas dos adolescentes de escolas estaduais de Curitiba, Paraná, em relação à hepatite A.

## Métodos

### Delineamento do estudo

Estudo transversal realizado entre os dias 3 e 4 de julho de 2024 incluindo cinco escolas estaduais do município de Curitiba, Paraná (denominadas Escola A, Escola B, Escola C, Escola D e Escola E). 

### Contexto

No período de 1/11/2023 a 29/5/2024, foram confirmados 281 casos de hepatite A em Curitiba, Paraná. Os primeiros registros concentraram-se em jovens adultos, mas ao longo da progressão do surto foram observados casos em crianças e adolescentes em idade escolar, ampliando o perfil etário afetado. Nesse contexto, esta investigação foi conduzida por uma equipe do Programa de Treinamento em Epidemiologia Aplicada aos Serviços do Sistema Único de Saúde (EpiSUS-Avançado), em parceria com a equipe de vigilância local.

### Participantes

O estudo foi conduzido com adolescentes de 16 anos ou mais, matriculados nas cinco escolas estaduais da rede pública de Curitiba, Paraná, selecionadas. Os critérios de elegibilidade foram:

- Inclusão: Participantes com idade de 16 anos ou mais que estavam regularmente matriculados e forneceram o consentimento livre e esclarecido para participação no estudo.
- Exclusão: Adolescentes cuja capacidade cognitiva não permitisse entender o questionário, conforme avaliação e informação fornecidas pelos responsáveis nas escolas.

Após a informação pela escola de quais os alunos elegíveis (≥16 anos) e da obtenção do consentimento, os participantes foram encaminhados para uma sala reservada na respetiva escola, onde receberam os esclarecimentos finais e preencheram o questionário de forma autoaplicável e anônima usando tablets fornecidos pela equipe, de modo a garantir o sigilo e a confidencialidade das respostas.

### Variáveis

O questionário contemplou variáveis sociodemográficas, de conhecimento e de práticas comportamentais relacionadas à hepatite A. As variáveis sociodemográficas incluíram: sexo, idade, raça/cor e escola de origem. As variáveis de conhecimento foram organizadas em três dimensões: i) Patologia - ter ouvido falar sobre hepatite A, tipo de doença (transmissível ou não), microrganismo causador e principal órgão afetado; ii) Transmissão e sintomatologia - modo de transmissão, sinais e sintomas, e exposições de risco; iii) Prevenção e cura - disponibilidade de vacina, formas de prevenção, possibilidade de cura e ocorrência prévia de infecção por hepatite A. As variáveis de práticas comportamentais foram agrupadas em cinco dimensões: i) Higiene - hábitos de lavagem das mãos e compartilhamento de objetos pessoais ou utensílios; ii) Hábitos alimentares - consumo de alimentos crus ou malpassados; iii) Água para consumo - origem e forma de tratamento da água, bem como hábitos relacionados ao uso de bebedouros escolares; iv) Produtos fumígenos - uso de cigarros ou outros produtos fumígenos; v) Práticas sexuais - iniciação sexual, características dos parceiros, uso de preservativo, sexo oro-anal, penetração anal (com ou sem preservativo), e penetração anal com dedos ou objetos.

O questionário foi pré-testado com técnicos da vigilância local antes de ser aplicado na ação de vigilância. Os investigadores optaram por focar nas dimensões de Conhecimentos e Práticas, que apresentam maior objetividade e reprodutibilidade, permitindo uma avaliação mais consistente do comportamento em relação à hepatite A. A dimensão Atitudes não foi analisada por se tratar de um construto subjetivo e de difícil mensuração em inquéritos autoaplicáveis, especialmente entre adolescentes.

### Fontes de dados e mensuração

Dados foram coletados através de um questionário semiestruturado autopreenchível disponibilizado na plataforma REDCap. Ele foi elaborado com base em estudos de conhecimentos, atitudes e práticas sobre hepatites virais ^6^ ^-^ ^10^ e no Guia de Vigilância em Saúde. A abordagem dos participantes foi realizada por intermédio das escolas selecionadas.

Para o cálculo dos escores de conhecimento, cada acerto equivalia a 1 ponto. O escore por dimensão foi transformado em uma escala decimal de 0 a 10 e o escore total foi igualmente padronizado, utilizando a pontuação total de acertos dividida pela pontuação máxima possível e multiplicada por 10. A distribuição dos pontos por dimensão de conhecimento é detalhada no link do item ‘disponibilidade de dados’. A pontuação das práticas foi atribuída em ordem crescente de risco, e o escore por dimensão e o escore total chamado de práticas de risco foram transformados em escala decimal de 0 a 10, em que pontuações mais altas indicavam maior risco. As pontuações por dimensão de práticas são detalhadas no link do item “disponibilidade de dados”. 

A classificação dos escores de conhecimento como a dos de práticas de risco seguiu as categorias: muito baixo (0 ⊢ 2,5), baixo (2,5 ⊢ 5), moderado (5 ⊢ 7,5) e alto (7,5 ⊢ 10). Escores mais altos em práticas indicam maior risco. Os escores foram calculados pela fórmula:

$$EC=\frac{Pontuação\;total\;no\;teste\;de\;conhecimento}{Pontuação\;máxima\;possível\;no\;teste}\times 10$$

### Vieses

Foram considerados como potenciais fontes de viés os de seleção, informação e classificação. O viés de seleção pode ter ocorrido em função da amostragem por conveniência e do período pré-férias escolares que implicou a redução de alunos nas escolas, limitando a generalização dos resultados. O viés de informação é inerente a estudos com questionário autoaplicável incluindo conteúdos sensíveis, podendo gerar viés de desejabilidade social (superestimação do conhecimento e subestimação de práticas de risco), o que foi mitigado pela garantia de anonimato. 

### Tamanho do estudo

Foram selecionadas cinco escolas por conveniência: quatro delas por terem registrado casos confirmados de hepatite A entre 1º de novembro de 2023 e 29 de maio de 2024, e uma (Escola E), sem casos confirmados, foi incluída para comparação. A Escola E situa-se em distrito sanitário na região sul de Curitiba caracterizado por indicadores socioambientais menos favoráveis, nomeadamente água e saneamento ^11^. 

A amostra estimada foi de 284 alunos, considerando uma frequência esperada de 50% e erro de 5%, com base em uma população de 1.090 estudantes com 16 anos ou mais. Os participantes foram selecionados por conveniência - todos os alunos elegíveis foram convidados a participar.

### Métodos estatísticos

Foi realizada análise descritiva das variáveis e comparações entre grupos por meio dos testes t de Student, Mann-Whitney e Kruskal-Wallis, com ajuste de comparações múltiplas pelo método de Bonferroni. As correlações entre variáveis contínuas foram avaliadas pelo coeficiente de Spearman. Foi adotado nível de significância de 5% (p-valor<0,05). As análises estatísticas foram conduzidas no software R (versão 4.4.0). 

## Resultados

Foram submetidos 300 questionários, dos quais 298 estavam preenchidos e incluídos na análise. A maioria dos participantes era do sexo feminino (52,0%) e raça/cor da pele branca (63,1%), com média de idade de 16,6 ± 0,7 anos (Tabela 1).

**Tabela 1:** Características demográficas dos participantes do estudo de conhecimentos e práticas sobre hepatite A. Curitiba, Paraná, 2024 (n=298)

Característica	n (%)
Sexo	
Feminino	153 (52,0)
Masculino	141 (48,0)
Raça/cor	
Branca	183 (63,1)
Parda	76 (26,2)
Preta	22 (7,6)
Amarela	5 (1,7)
Indígena	4 (1,4)
Escolas	
Escola A	50 (16,8)
Escola B	73 (24,5)
Escola C	47 (15,8)
Escola D	42 (14,1)
Escola E	73 (24,5)

A avaliação do conhecimento revelou que, embora uma parte significativa dos adolescentes (70,1%) já tenha ouvido falar sobre a hepatite A, o conhecimento específico sobre a doença foi limitado (Tabela 2). 

O escore médio geral de conhecimento sobre hepatite A foi de 3,7±3,0, correspondendo à classificação de conhecimento baixo. Houve maior pontuação entre estudantes do sexo feminino (p-valor 0,039) (Tabela 3). Não houve diferença estatisticamente significativa nos escores de conhecimento entre as escolas (p-valor 0,258).

A dimensão Patologia apresentou melhores resultados em relação à “transmissão e sintomatologia” (p-valor<0,001), como observado na Tabela 4. A menor proporção de acertos foi observada nas questões sobre exposições de risco, formas de prevenção e sinais e sintomas da doença.

**Tabela 2:** Conhecimentos específicos sobre hepatite A entre adolescentes escolares segundo tema, questão e resposta correta. Curitiba, Paraná, 2024 (n=298)

Tema	Questão abreviada	Resposta correta (%)
Conhecimento geral	Já ouviu falar em hepatite A?	Sim (70,1)
Transmissibilidade	Tipo de doença	Transmissível (43,6)
Agente etiológico	Causada por	Vírus (27,9)
Forma de infecção	Principal via	Fecal-oral (39,9)
Órgão acometido	Qual órgão?	Fígado (30,2)
Vacina	Existe vacina?	Sim (46,6)
Exposição de risco	Água/alimentos contaminados	Sim (36,2)
	Sexo oral-anal sem proteção	Sim (35,2)
Sinais/sintomas	Febre	Sim (42,3)
	Olhos amarelados	Sim (35,2)
Prevenção	Higienização das mãos	Sim (39,9)
Cura	É curável?	Sim (40,3)

**Tabela 3:** Escores de conhecimento sobre hepatite A por sexo, escolas e dimensões de conhecimento, apresentados como média (±DP) e mediana (IIQ). Curitiba, Paraná, 2024 (n=298)

Variáveis	Média (±DP)	Mediana (IIQ)	p-valor
Geral	3,7 (±3,0)		
Sexo			
Feminino	4,0 (±3,1)	4,7 (6,5)	0,039^a^
Masculino	3,3 (±2,9)	4,1 (5,9)	
Escolas			
Escola C	4,3 (±2,9)	4,7 (4,4)	0,258^b^
Escola D	4,0 (±3,1)	4,7 (5,9)	
Escola E	3,8 (±2,9)	4,7 (6,5)	
Escola B	3,8 (±3,0)	4,1 (6,5)	
Escola A	2,9 (±2,9)	2,7 (4,7)	
Dimensão de conhecimento			
Patologia	4,3 (±3,5)	5,0 (7,5)	0,004^c^
Transmissão e sintomatologia	3,3 (±3,2)	3,8 (6,3)	
Prevenção e cura	3,8 (±3,5)	4,0 (6,0)	

^a^ Teste t de Student; ^b^Teste Anova; ^c^Teste de Kruskal-Wallis.

**Tabela 4:** Escores de práticas de risco sobre hepatite A por sexo, escolas e dimensões, apresentados como média (±DP). Curitiba, Paraná, 2024 (n=298)

Variáveis	Média (±DP)	p-valor
Geral	1,7 (±1,3)	
Sexo		
Masculino	1,9 (±1,5)	0,001^a^
Feminino	1,4 (±0,9)	
Escolas		
Escola D	2,1 (±1,4)	0,016^b^
Escola B	1,8 (±1,4)	
Escola C	1,7 (±1,3)	
Escola A	1,6 (±1,1)	
Escola E	1,3 (±1,1)	
Dimensão de práticas		
1. Higiene	2,7 (±1,5)	<0,001^b^
2. Hábitos alimentares	3,9 (±4,4)	
3. Produtos fumígeno	1,3 (±1,3)	
4. Água para consumo	2,5 (±3,4)	
5. Práticas sexuais	6,3 (±1,4)	

^a^ Teste t de Student; ^b^Teste de Kruskal-Wallis.

Quanto às práticas de risco, o escore médio foi de 1,7 ± 1,3, classificado como escore de práticas de risco muito baixo. Estudantes do sexo masculino apresentaram maior risco (p-valor 0,001). Destacaram-se escores mais altos nas dimensões “práticas sexuais” e “hábitos alimentares”. Houve diferença significativa entre escolas (p-valor 0,016), especificamente entre a Escola E e a Escola D (p-valor 0,010; teste u de Mann-Whitney) (Tabela 4). 

Não foi observada correlação entre os escores de conhecimento e de práticas de risco (correlação de Spearman rho=0,00; p-valor 0,983).

## Discussão

Os achados deste estudo revelam que, embora a hepatite A seja uma patologia nominalmente familiar à maioria dos adolescentes das escolas avaliadas, existe uma lacuna de conhecimento especialmente no que tange aos mecanismos de transmissão e ao reconhecimento de sinais e sintomas. Esse perfil de conhecimento contrasta com um padrão de práticas de risco predominantemente reduzido, sugerindo que o comportamento preventivo no grupo estudado pode ser mediado por hábitos cotidianos e fatores socioambientais, em vez de uma decisão instruída sobre o agravo. 

Entre os pontos fortes do estudo, destaca-se a oportunidade de obtenção de dados em contexto de surto recente, obtidos de um instrumento detalhado e padronizado. No entanto, algumas limitações devem ser consideradas. A amostragem por conveniência de cinco escolas e a inclusão apenas de adolescentes com 16 anos ou mais limitam a generalização dos achados a todas as escolas de Curitiba. A coleta no período pré-férias pode ter reduzido a adesão, configurando possível viés de seleção. Além disso, o autorrelato pode ter introduzido viés de desejabilidade social, ainda que minimizado pelo preenchimento anônimo do questionário. Deve-se também reconhecer a ausência de ajuste para possível efeito de conglomerado entre escolas, bem como a não inclusão do componente “Atitudes” no instrumento, o que restringe a análise a conhecimentos e práticas e limita a compreensão dos determinantes comportamentais subjacentes.

O escore médio de práticas de risco foi classificado como muito baixo, o que pode refletir tanto práticas efetivamente mais seguras (baixa exposição a comportamentos de risco) como possível viés de desejabilidade social nas respostas. No entanto, a dimensão “Práticas Sexuais” apresentou pontuação mais elevada em comparação às demais. No contexto da hepatite A, esse achado é epidemiologicamente relevante sobretudo em relação a práticas oro-anais e de higiene pessoal relacionadas, reconhecidos como possíveis vias de transmissão fecal-oral. 

Foi observado que adolescentes do sexo feminino apresentaram um escore de conhecimento significativamente maior que o sexo masculino, padrão que é consistente com a literatura ^12^ ^,^ ^13^. Apesar dessa diferença, o escore geral de conhecimento baixo alinha-se aos achados de estudos sobre hepatites virais em adolescentes e jovens adultos ^6^ ^,^ ^7^ ^,^ ^10^. O achado de maiores práticas de risco no sexo masculino também está em consonância com estudos prévios que indicam maior prevalência de práticas de risco entre meninos, especialmente em relação ao uso inconsistente de preservativos e início da vida sexual mais cedo ^14^. A ausência de correlação entre conhecimento e práticas de risco também já foi descrita em outras pesquisas de conhecimentos, atitudes e práticas (CAP), indicando que a informação isolada pode não ser suficiente para modificar comportamentos, especialmente entre adolescentes, cujas atitudes são fortemente influenciadas por pares, cultura e fatores psicossociais ^15^ ^,^ ^16^. No geral, a ocorrência de casos em adolescentes durante o surto em Curitiba, Paraná, e o alto escore de risco nas práticas sexuais nessa faixa etária apontam para uma vulnerabilidade que exige atenção, considerando não apenas o risco à infecção por hepatite, mas a outras de transmissão sexual, como o Vírus da Imunodeficiência Humana (HIV), em crescimento entre os jovens ^17^.

Este estudo identificou lacunas significativas de conhecimento sobre a hepatite A entre adolescentes escolares e presença de práticas de risco elevadas no domínio da sexualidade. Esses achados indicam a relevância de considerar o nível de informação e os comportamentos relacionados à saúde no planejamento de ações voltadas a esse grupo etário.

## Data Availability

Os bancos de dados anonimizados empregados neste estudo estão disponibilizados publicamente no repositório SciELO Data, vinculado à revista *Epidemiologia e Serviços de Saúde* (RESS), e podem ser acessados pelo link: https://doi.org/10.48331/SCIELODATA.6J5S6L. A utilização do conjunto de dados deve ser acompanhada da citação conforme a referência indicada no próprio repositório.
